# Influence of Scribble polarity complex on hematopoiesis and leukemia – a matter of where, when and how

**DOI:** 10.18632/oncotarget.26132

**Published:** 2018-10-05

**Authors:** Florian H. Heidel, Sarah Ellis

**Affiliations:** Innere Medizin II, Hämatologie und Onkologie, Universitätsklinikum Jena, Am Klinikum 1, Jena, Germany; Leibniz Institute on Aging, Fritz-Lipmann Institute, Jena, Germany; Peter MacCallum Cancer Centre, Melbourne, Victoria, Australia; Sir Peter MacCallum Department of Oncology, The University of Melbourne, Parkville, Victoria, Australia

**Keywords:** Scribble, Scrib, Llgl1, polarity, stem cell

A key characteristic of hematopoietic stem cells (HSC) is their ability to self-renew. Several evolutionarily conserved genes and pathways control the fine balance between self-renewal and differentiation in HSC and potentially also in leukemic stem cells (LSC). *In vivo* RNAi screens have identified polarity regulators that enhanced (e.g. Prox1) or diminished (e.g. Pard6a, Prkcz, Msi2) the repopulation potential of HSCs *in vivo* [1]. However, few of these genes and proteins have been investigated in detail.

The Scribble complex, which was discovered and characterized in *Drosophila melanogaster*, consists of three proteins: Scribble (Scrib), Discs large (Dlg) and Lethal giant larvae (Lgl). These complex members serve as scaffolding proteins and regulate cell polarity, motility and growth mainly through protein–protein interactions. In *Drosophila*, genetic inactivation of single Scribble complex members leads to neoplastic tissue overgrowth supporting the description of these proteins as one of the earliest tumor suppressors [2]. Recent data suggests the involvement of Scribble complex proteins in the regulation of HSC biology with potential implications in leukemia development. In mammalian hematopoiesis, several homologues of Llgl and Dlg have been identified (Llgl 1&2 and Dlg1-4) whilst Scrib exists as a single homologue.

Genetic inactivation of *Llgl1* is associated with a significant increase in long-term (LT-) HSC numbers and these cells show a competitive advantage when transplanted serially into recipient mice [3]. Llgl1 deletion by itself does not cause leukemia, however, decreased expression of Llgl1 correlates with decreased survival in AML patients. Recently, mutations in human *Llgl2*, have been described as an early genetic event in the progression from severe congenital neutropenia to AML [4]. In contrast, the tumor suppressor function of Llgl1 is not conserved in murine models of lymphoid (B- and T-cell) leukemia [5] suggesting Llgl acts in a cell context specific manner.

Less data is available on the function of Dlg in hematopoiesis and leukemic transformation. Dlg is important for the immunological synapse formation in mature T cells. Conditional inactivation of Dlg in murine hematopoiesis disturbs the maturation process of B lymphocytes, specifically in a novel stage of pre-B cells marked by expression of c-Myc [6]. Moreover, inactivation of Dlg promotes the development of BCR-ABL and p53 driven B cell leukemia/lymphoma [6], indicating a conserved role of Dlg as a tumor suppressor in the B-lymphoid lineage.

Similarly to Lgl1, the influence of Scrib on lymphoid cells is cell context dependent. Whilst Scrib is not required for mature B-cell function and humoral immunity [7], its inactivation affects immature T-cell polarization and function [8].

In their recent paper, Mohr and colleagues revealed loss of self-renewal in adult murine HSCs following genetic inactivation of Scrib [9]. Comparable to the role of Llgl1 in HSC function, this effect was restricted to LT-HSC. While steady state hematopoiesis was not affected, serial transplantation and application of cell stress resulted in impaired HSC function. In a second report, the authors provided evidence that Scrib-deficient hematopoietic stem- and progenitor cells (HSPCs) fail in proper adhesion and have decreased migratory capacity [10], effects that were not detected in HSPCs after inactivation of Llgl1. With regards to malignant transformation, deletion of Scrib impaired the development of AML *in vivo* and this negative effect on LSC function was underlined by selection of partially and non-excised clones in secondary recipient hosts. Whilst these findings are in contrast to the role of Scrib in *Drosophila* and to its well-described tumor-suppressor role in epithelial cells, a requirement for Scrib in lymphomagenesis [11] has been confirmed recently. Here, loss of Scribble expression delayed the expansion of B-cells and the onset of Eμ-myc driven Burkitt's lymphoma.

While specific cellular functions of Scribble complex members appear to be conserved in mammalian hematopoiesis, they appear to be highly context dependent. Depending on the differentiation stage (stem cell, committed progenitor or mature blood cell), lineage (myeloid, B- or T-lymphoid), underlying genetic background (co-existing genetic mutations or oncogenes), setting of inactivation (conventional versus conditional inactivation; gene dosage) and cellular state (quiescent versus activated/stressed), functional consequences in hematopoietic cells may be highly variable and phenotypically diverse. This needs to be taken into consideration when comparing published model systems and specifically hematopoietic neoplasms.

Taken together, recent data confirm a distinct role for Scribble complex members in various model systems and underline their impact on normal hematopoiesis and malignant transformation.
